# Acquired von Willebrand syndrome and factor VIII in patients with moderate to severe mitral regurgitation undergoing transcatheter mitral valve repair

**DOI:** 10.1002/clc.23538

**Published:** 2020-12-29

**Authors:** Christine Meindl, Michael Paulus, Theresia Koller, Dominik Rogalski, Michael Hamerle, Christian Schach, Stefan Buchner, Florian Zeman, Lars S. Maier, Kurt Debl, Bernhard Unsöld, Christoph Birner

**Affiliations:** ^1^ Department of Internal Medicine II University Hospital Regensburg Regensburg Germany; ^2^ Department of Internal Medicine II Sana Hospital Cham Cham Germany; ^3^ Center for Clinical Studies University Hospital Regensburg Regensburg Germany; ^4^ Department of Internal Medicine I St. Marien Hospital Amberg Amberg Germany

**Keywords:** MitraClip, mitral valve repair, PASCAL, von Willebrand factor

## Abstract

**Background and Hypothesis:**

The acquired von Willebrand syndrome (AvWS), which predisposes to bleeding events, is often related to valvular heart diseases. We investigated possible implications of AvWS and factor VIII levels in patients with moderate to severe mitral regurgitation (MR) undergoing transcatheter mitral valve repair (TMVR).

**Methods and Results:**

123 patients with moderate to severe MR were prospectively enrolled. Complete measurements of von Willebrand Factor activity (vWFAct), von Willebrand Factor antigen (vWFAg), and factor VIII expression before and 4 weeks after TMVR were available in 85 patients. At baseline, seven patients had a history of gastrointestinal bleeding, two patients suffered bleeding events during their hospital stay, and one patient had a bleeding 4 weeks after TMVR. Even though vWFAct, vWFAct/vWFAg ratio and vWFAg values did not change after TMVR, we observed a significantly lower vWFAct/vWFAg ratio in patients with primary MR as compared to patients with secondary MR both at baseline (p = 0.022) and 4 weeks following the TMVR procedure (p = 0.003). Additionally, patients with a mean mitral valve gradient ≥4 mmHg after TMVR had significantly lower vWFAct/vWFAg ratios as compared to patients with a mean mitral valve gradient <4 mmHg (p = 0.001).

**Conclusions:**

MR of primary etiology was associated with lower vWFAct/vWFAg ratio, hinting toward HMWM loss due to shear stress caused by eccentric regurgitation jets. In addition, morphological changes leading to postprocedural transmitral gradients ≥4 mmHg were related to lower vWFAct/vWFAg ratio 4 weeks after the procedure. Alterations of the vWFAct/vWFAg ratio in turn did not translate into a greater risk for bleeding events.

## 1 INTRODUCTION

Von Willebrand factor (vWF) is produced in megakaryocytes and endothelial cells and functions as a large human adhesive glycoprotein.^1^, ^2^

Inherited von Willebrand Disease (vWD) represents the most common inherited bleeding disorder, which was first described by Erik von Willebrand in 1926.^3^ In contrast to the inherited form, acquired von Willebrand syndrome (AvWS) is a rare disorder^4^ with an estimated prevalence of 0.04%, although this figure might be underestimated.^5^ AvWS is often associated with cardiovascular diseases especially congenital and valvular heart diseases.^6^ AvWS is similar to inherited type 2A vWD showing decreased vWF‐dependent platelet adhesion due to a loss of high‐molecular‐weight von Willebrand Factor multimers.^7^

Heyde syndrome comprises the combination of AvWS, valvular heart disease and bleeding tendency – in particular gastrointestinal bleeding‐^8^ as a result of an increased clearance of vWF related to shear‐induced proteolysis of high‐molecular‐weight vWF multimers when passing the abnormal valve.^9^, ^10^

High‐molecular‐weight vWF multimer abnormalities have been reported in patients with different valvular diseases e.g. aortic and mitral valve stenosis as well as mitral valve regurgitation.^11^ VWF deficiency can be completely reversed after aortic valve replacement therapy^12^ thus, vWF has been proposed as a new hemodynamic biomarker for monitoring surgical or percutaneous aortic valve replacement procedures.^13^, ^14^, ^15^

It has been reported that defects of high‐molecular‐weight vWF multimers were resolved within minutes during transcatheter aortic valve replacement (TAVR) but not after ballon valvuloplasty procedures.^16^

Similarly, the presence of AvWS has been described in patients with moderate to severe mitral valve dysfunction.^1^ Blackshear et al. investigated a population of 53 patients with mild to severe mitral regurgitation and reported that more severe mitral valve regurgitation was associated with progressive worsening of vWF activity and bleeding tendency.^17^ Lower levels of high‐molecular‐weight vWF multimers were associated with poor prognosis concerning surgery‐free survival.^17^

In contrast to patients with aortic valve stenosis undergoing TAVR only little data exist regarding vWF function in patients with mitral valve regurgitation treated by transcatheter mitral valve repair (TMVR). We therefore investigated AvWS and factor VIII in a large cohort of patients with moderate to severe mitral regurgitation undergoing a transcatheter mitral valve repair.

## 2 METHODS

### 2.1 Study population

From August 2017 to May 2020 123 patients with symptomatic moderate to severe or severe mitral valve regurgitation were prospectively enrolled in our investigator initiated single‐center RETORT‐MR trial (Regensburg Trial on TMVR Techniques in Mitral Regurgitation). All patients gave informed consent before enrollment and the RETORT‐MR trial was approved by the local ethics committee. No external funding was obtained to support the study. Comprehensive measurements of von Willebrand Factor activity (vWFAct), von Willebrand antigen (vWFAg), vWFAct/vWFAg ratio and factor VIII before and 4 weeks after the percutaneous mitral valve repair were available in 85 patients.

Clinical data were collected from all participants and serial blood and urine sampling was performed. In addition, transthoracic (TTE) and transesophageal (TOE) echocardiography was conducted. Special attention was paid to history of bleeding events and antiplatelet or anticoagulant medication. EuroScore II and logEuroScore were calculated to assess operative risk.^18^

After discussion of every case in the interdisciplinary heart team 83 of the included patients underwent transcatheter mitral valve repair using the MitraClip NT, NTR, or XTR system, Abbott Vascular (Menlo Park, CA) and two patients received PASCAL devices, Edwards Lifesciences (Irvine, CA).

### 2.2 Transcatheter mitral valve repair

Technical aspects of the MitraClip^19^ and PASCAL^20^ procedure have been described previously.

All patients were under general anesthesia during the procedure and both fluoroscopic and transesophageal echocardiographic guidance were used for percutaneous mitral valve repair. During the procedures, one to three MitraClips and one or two PASCAL devices were implanted in order to achieve a significant reduction of mitral regurgitation. Heparin was administered for periprocedural anticoagulation. After percutaneous mitral valve repair patients received dual antiplatelet therapy consisting of aspirin and clopidogrel for 4 weeks, followed by aspirin administration for 6 months. In case patients had already been treated with oral anticoagulants, aspirin or clopidogrel was added for 1 month.

### 2.3 Laboratory analysis

Von Willebrand Factor activity, von Willebrand Factor antigen and factor VIII were measured at baseline and after 4 weeks and results at both points of time were available in 85 patients. Von Willebrand Factor activity was analyzed using the INNOVANCE® VWF Ac system provided by SIEMENS (Siemens Healthcare GmbH, Erlangen/Germany), and von Willebrand Factor antigen was measured by immunoturbidimetry using the measuring devices of Siemens Healthcare Diagnostics or SYSMEX (SYSMEX CORPORATION). The results of factor VIII were gained by in‐vitro diagnostics of coagulation factor VIII deficient plasma by SIEMENS (Siemens Healthcare GmbH, Erlangen/Germany).

### 2.4 Clinical follow‐up

Clinical assessment including bleeding history, standardized echocardiographic parameters and serial blood and urine sampling was performed 24 hours before and 4 weeks after percutaneous mitral valve repair. In addition, six‐minute walk tests and the EQ‐5D‐5L questionnaire were performed to investigate patients' physical and mental status.

Two‐dimensional transthoracic echocardiography was conducted using the iE‐33 ultrasound system with a S5‐1 transducer (Philips Medical Systems, Amsterdam, The Netherlands) or the EPIQ CVx ultrasound system with a X5‐1 transducer (Philips Medical Systems, Amsterdam, The Netherlands). The quantification of mitral regurgitation was in accordance with the Endovascular Valve Edge‐to‐Edge Repair Study (EVEREST) criteria.^21^

### 2.5 Statistical analysis

Statistical analysis was performed with SPSS Version 25 (International Business Machines Corporation, IBM, Armonk, NY USA). Data are presented as absolute and relative frequencies or as means ± standard deviations. Differences between continuous variables in paired data were tested with a paired t‐test, continuous variables in unpaired data were compared with an unpaired t‐test. Analysis of variance was used to compare continuous variables between more than two independent groups. For evaluating differences in unpaired data adjusted for covariates, analysis of covariance was performed. A *p‐*value of <0.05 was considered statistically significant.

## 3 RESULTS

Baseline characteristics of the study population are depicted in Table 1. The mean patients' age was 75.9 ± 8.4 years and 58.8% of patients were men. In 24 patients (28.2%) primary mitral regurgitation was diagnosed, whereas 55 (64.7%) suffered from secondary mitral regurgitation. Six patients (7.1%) showed a combined etiology. The majority of patients had severe comorbidities such as coronary artery disease (58.8%), anemia (38.8%) or chronic kidney disease (85.9%). 55.3%, 17.6%, and 27.1% had an ejection fraction ≥50%, an ejection fraction 40–49%, and an ejection fraction <40%, respectively.

**TABLE 1:** Baseline characteristics of the study population (n = 85)

Age, yrs, mean ± SD	75.9 ± 8.4
Male gender, n (%)	50 (58.8)
BMI, kg/m^2^, mean ± SD	26 ± 4.2
Primary MR, n (%)	24 (28.2)
Secondary MR, n (%)	55 (64.7)
Combined MR, n (%)	6 (7.1)
MR grade III, n (%)	18 (21.2)
MR grade IV, n (%)	67 (78.8)
MV mean gradient, mmHg±SD	2.48 ± 1.1
Ejection fraction <40%, n (%)	23 (27.1)
Ejection fraction 40–49%, n (%)	15 (17.6)
Ejection fraction ≥50%, n (%)	47 (55.3)
LVEF, % ± SD	46.5 ± 15.2
Arterial hypertension, n (%)	61 (71.8)
Atrial fibrillation, n (%)	54 (63.5)
Dilated cardiomyopathy, n (%)	8 (9.4)
Coronary artery disease, n (%)	50 (58.8)
Anemia, n (%)	33 (38.8)
Chronic kidney disease, n (%)	73 (85.9)
Diabetes mellitus, n (%)	24 (28.2)
History of gastrointestinal bleeding, n (%)	7 (8.2)
ICD, n (%)	14 (16.5)
NYHA class II, n (%)	5 (5.9)
NYHA class III, n (%)	76 (89.4)
NYHA class IV, n (%)	4 (4.7)

Abbreviations: BMI, body mass index; ICD, implantable cardioverter defibrillator; LVEF, left ventricular ejection fraction; MR, mitral regurgitation; MV, mitral valve; n, number; NYHA, New York Heart Association; SD, standard deviation; Yrs, years.

**FIGURE 1:**
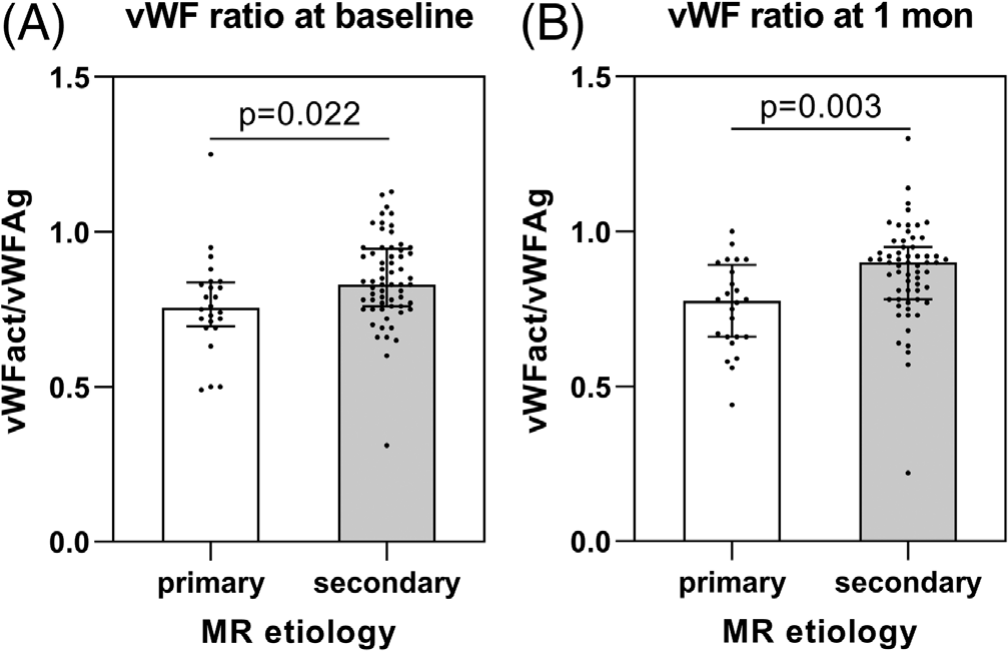
vWFAct/vWFAg ratio at baseline (1A) and 4 weeks follow‐up in respect of MR etiology

**FIGURE 2:**
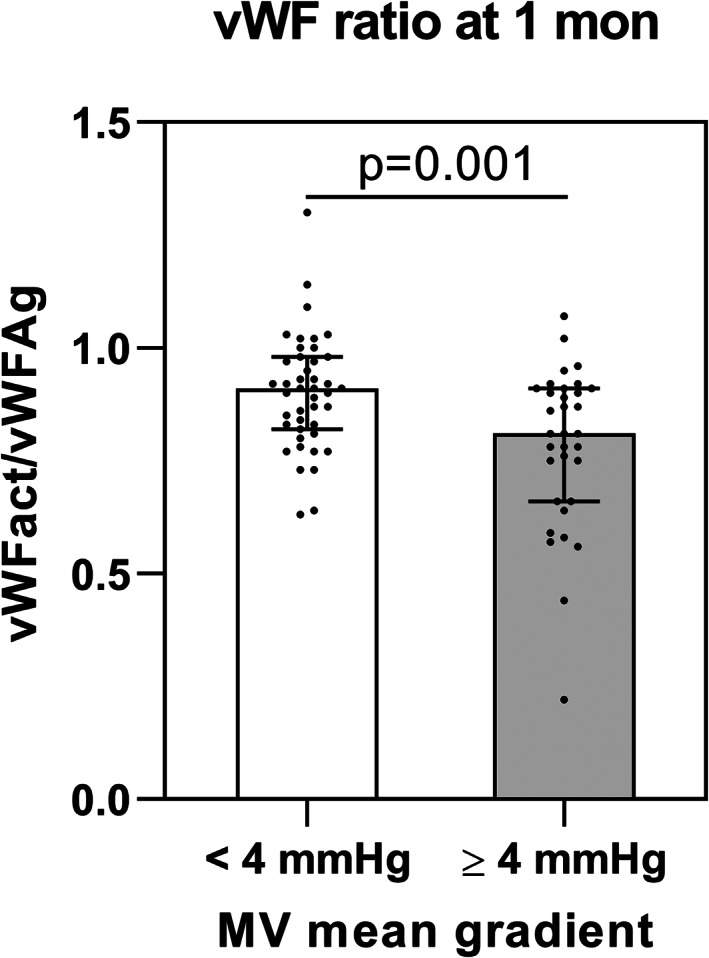
vWFAct/vWFAg ratio at 4 weeks follow‐up in respect of MV mean gradient

The frequency of gastrointestinal bleeding at baseline was 8.2% (n = 7). Two in‐hospital bleedings (Table 2) occurred (2.4%) and at 4 weeks follow‐up another bleeding (1.2%) was reported (Table 3). It is noteworthy that the patient suffering from the in‐hospital bleeding already had had a history of gastrointestinal bleeding at baseline. Concerning medication this patient was treated with aspirin but with no oral anticoagulant. The documented bleeding case 4 weeks after the MitraClip procedure occurred under dual antiplatelet therapy with aspirin and clopidogrel. Antiplatelet and oral anticoagulation medication is depicted in detail in Table S1 in the supporting information. Table 3 summarizes the clinical and echocardiographic results at 4 weeks follow‐up with reduction of mitral regurgitation to grade I or II in 88.2% of patients. Table S2 shows the levels of vWFAct, vWFAg, vWFAct/Ag ratio and factor VIII at baseline and 4 weeks after the TMVR procedure. No significant changes related to the procedure could be observed. However, patients with primary MR had significantly lower vWFAct/vWFAg ratios as compared to patients with secondary MR at baseline (p = 0.022) and at 4 weeks follow‐up (p = 0.003) (Figure 1(A) and (B)). In addition, patients with a postprocedural mean mitral valve gradient ≥4 mmHg had significantly lower vWFAct/vWFAg ratios as compared to patients with a mean mitral valve gradient <4 mmHg (p = 0.001) (Figure 2).

**TABLE 2:** Postprocedural results of the study sample

Device type, n (%)	NT	43 (50.6)
	NTR	26 (30.6)
	XTR	5 (5.9)
	XTR&NTR	9 (10.6)
	PASCAL	2 (2.4)
Number of implanted devices	1	45 (52.9)
	2	38 (44.7)
	3	2 (2.4)
Mitral valve surgery, n (%)		1 (1.2)
Device embolization, n (%)		0
Leaflet detachment, n (%)		3 (3.5)
Valve lesion, n (%)		2 (2.4)
Pericardial tamponade, n (%)		1 (1.2)
In hospital stroke, n		0
In hospital bleeding, n (%)		2 (2.4)
In hospital death, n (%)		1 (1.2)

**TABLE 3:** Patient characteristics 4 weeks after TMVR

MR grade I, n (%)	47 (55.3)
MR grade II, n (%)	28 (32.9)
MR grade III, n (%)	4 (4.7)
MR grade IV, n (%)	2 (2.4)
NYHA class I, n (%)	20 (23.5)
NYHA class II, n (%)	47 (55.3)
NYHA class III, n (%)	16 (18.8)
MV mean gradient, mmHg±SD	3.6 ± 1.4
LVEF, % ± SD	46.2 ± 14.8
Major bleeding since TMVR, n (%)	1 (1.2)

After adjustment for age and sex the results of vWFAct/vWFAg remained stable concerning MR etiology (p‐value = 0.013) and MV gradient ≥4 mmHg (p‐value = 0.001).

By stratifying the cohort into low and high post mitral valve gradients (≥4 mmHg versus <4 mmHg), no significant results were found between the groups at baseline and 4 weeks follow‐up (Table S3). Further, we analyzed the values of vWFAct/vWFAg, vWFAg, vWFAct and Factor VIII at baseline and 4 weeks follow‐up with mild residual MR and low post MV gradient (Table S4). Again, no significant results were detected in those groups. This means that the lacking decrease of the analyzed parameters cannot sufficiently be explained by a sustained high transvalvular shear stress, which could be caused either by a TMVR‐induced increase of the MV gradient, or a residual mitral regurgitation.

Table S5 shows a subgroup analysis of vWFAct, vWFAg, vWFAct/vWFAg ratio and factor VIII levels of patients with primary MR at baseline and 4 weeks follow‐up and with secondary MR at baseline and 4 weeks follow‐up. In both groups no significant changes were revealed, which means that the expression of the analyzed parameters might not be etiology‐specific.

Further no significant differences in the absolute values of vWFAct/vWFAg, vWFAg, vWFAct and Factor VIII were observed (Table S6).

Residual mitral regurgitation after TMVR did not significantly influence vWFAct, vWFAg, vWFAct/vWFAg ratio and factor VIII levels (Table S7). The values of vWFAct, vWFAg, vWFAct/vWFAg ratio and factor VIII were also not influenced by the type of heart failure (EF≤50% vs. EF > 50%, Table S8). No significant differences regarding vWFAct, vWFAg, vWFAct/vWFAg ratio and factor VIII expression were detected between the different implanted devices (i.e., MitraClip NT&NTR vs. MitraClip XTR vs. PASCAL device, Table S9). As depicted in Table S10 in the supporting information also the number of clips did not have any significant impact on the values.

## 4 DISCUSSION

To the best of our knowledge, we investigated vWFAct, vWFAg, vWFAct/vWFAg ratio and factor VIII in the largest cohort of patients treated with transcatheter mitral valve repair so far. We were able to reveal significant differences in the values of vWFAct/vWFAg ratio at baseline and 4 weeks follow‐up in respect of MR etiologies and mean mitral valve gradients after percutaneous mitral valve repair.

### 4.1 vWFAct/Ag ratio is influenced by the MR etiology

In our study primary MR was associated with a significantly decreased vWFAct/vWFAg ratio compared to secondary MR indicating a HMWM loss. As stated by Chen et al. it is important to take the jet morphology into consideration.^22^ Usually in primary MR the regurgitant jet is eccentric, striking the left atrial wall close to the mitral annulus.^22^ These flow alterations may cause increased shear stress leading to HMWM loss.

vWF levels are influenced by different hemodynamic conditions. As characterized by Heyde syndrome AvWS, valvular heart disease and bleeding tendency result in an increased clearance of vWF due to shear stress leading to a proteolysis of HMWM vWF during the passage of the abnormal heart valve.^9^, ^10^ Blackshear et al. described in a selected cohort of patients that increasing MR severity was associated with progressive worsening of vWF activity and bleeding tendency.^17^ In this study, Heyde syndrome had been diagnosed in 13% of cases. A significant association of lower levels of HMW‐vWF multimers and a poorer prognosis in respect of surgery‐free survival was found. Both surgical mitral valve repair and replacement were associated with a significant increase in vWF function.^17^ Similar effects were shown in a small study investigating percutaneous mitral valve repair via MitraClip implantation.^1^

Interestingly, our distinctly larger RETORT‐MR trial could not confirm these results. Besides the considerably different sample sizes, which could have biased the results preferentially in smaller trials, the main reason for this finding could be the time when vWFAct and vWFAg were measured: Gragnano et al. analyzed vWFAct and vWFAg levels already 24 hours after percutaneous mitral valve repair,^1^ whereas our study presents the levels of vWFAct and vWFAg not earlier than 4 weeks after MitraClip implantation. We deliberately chose this point of time to avoid the influence of the TMVR procedure and general anesthesia on hemostasis as it is known that stress may cause fluctuation of vWF levels^23^ with unclear functional relevance. Insofar, our results might reflect the clinically more relevant long term effects of TMVR on vWFAct and vWFAg levels rather than a transient acute effect.

In patients with severe aortic valve stenosis undergoing transcatheter aortic valve replacement (TAVR) an acute recovery of HMWM‐vWF levels was observed within a few minutes after successful TAVR.^16^, ^24^ Thus our results might have been different if we had measured vWFAct and vWFAg immediately or only a few minutes after percutaneous mitral valve repair, albeit the functional relevance of merely transient fluctuations would at best be questionable. Further studies are needed to detect possible differences in vWF levels at various points of time.

### 4.2 vWFAct/Ag ratio is influenced by the postprocedural transmitral gradient

In terms of mitral valve stenosis only little data exist with regard to the AvWS. In a small study of Yetkin et al, higher plasma levels of vWF were found in patients with mitral stenosis (n = 21) compared to a control group.^25^

In our study patients with a mean mitral valve gradient ≥4 mmHg after percutaneous mitral valve repair had significantly lower vWFAct/vWFAg ratios compared to patients with a mean mitral valve gradient <4 mmHg. Both the MitraClip and the PASCAL device represent foreign material with possible effects on hemostasis. Furthermore, the geometry of the mitral valve is changed by percutaneous mitral valve repair, which may cause abnormal transvalvular flow patterns, resulting in regional hemodynamic shear stress.

Facing the MV cut‐off gradients derived from our study, it is noteworthy, that Neuss et al. found a negative effect on the overall clinical outcome in their study, when the mean postprocedural mitral valve gradient exceeded 4.4 mmHg.^25^ Unfortunately, this study did not further specify the clinical patient outcomes, especially regarding bleeding events.

Besides hemodynamic conditions it has also been shown by several studies that vWF levels are affected by age. In centenarians vWF values were significantly elevated.^26^, ^27^ This seems to be an important issue as the mean age of our study population was 75.9 years (±8.4 SD). In general, a shift toward a more procoagulant status is proposed with aging^28^, ^29^, ^30^ but data about the underlying mechanisms of the age dependent increase in vWF levels is lacking.^27^

### 4.3 Limitations

Some limitations of the present study warrant consideration: Our trial was performed at an experienced academic institution, which means that our results might not completely be transferable to less trained centers. Finally, due to the limited number of bleeding events in our study, we were not able to estimate the effect of percutaneous mitral valve repair on an overall bleeding diathesis. We demonstrated significant differences in the values of vWFAct/Ag ratio in respect of degenerative and functional MR as well of elevated postprocedural gradients. In order to investigate the underlying pathophysiological mechanisms like shear stress further experimental studies would be needed.

## 5 CONCLUSION

In this largest investigation of AvWS in patients with mitral regurgitation so far, performing TMVR did not alter vWF levels or vWF activity 4 weeks after the procedure. MR of primary etiology was associated with lower vWFAct/vWFAg ratio, hinting toward HMWM loss due to shear stress caused by eccentric regurgitation jets. In addition, postprocedural transmitral gradient ≥4 mmHg was related to lower vWFAct/vWFAg ratio 4 weeks after the procedure. Bleeding events in the short‐term after TMVR were rare despite high perioperative risk, confirming the superior safety profile of percutaneous mitral valve repair.

## CONFLICT OF INTEREST

The authors declare no potential conflict of interest.

## Supporting information


**Appendix** **S1: Supporting information**

## Data Availability

The data that support the findings of this study are available on request from the corresponding author.
