# Supplementary material for: Acquired von Willebrand syndrome and factor VIII in patients with moderate to severe mitral regurgitation undergoing transcatheter mitral valve repair
Source: Clin Cardiol. 2020 Dec 29;44(2):261–6. doi: 10.1002/clc.23538 (PMC7852171; doi:10.1002/clc.23538)
Supplement: Supplementary file 1 — Appendix S1: Supporting information [file CLC-44-261-s001.docx]

**Supplementary Appendix**

Table S1. Antiplatelet and oral anticoagulation medication

|  | Baseline | 4 weeks follow-up |
| --- | --- | --- |
| Aspirin, n (%) | 33 (38.8) | 44 (51.8) |
| P2Y12 inhibitor, n (%) | 22 (25.9) | 38 (44.7) |
| Vitamin K antagonist, n (%) | 22 (25.9) | 21 (24.7) |
| Direct oral anticoagulant, n (%) | 29 (34.1) | 31 (36.5) |

Table S2. Values of vWFAct, vWFAg, vWFAct/vWFAg, factor VIII at baseline and 4 weeks follow-up

|  | baseline | 4 weeks follow-up | p-value |
| --- | --- | --- | --- |
| vWFAct | 196.6 ± 79.3% | 194.6 ± 78.1% | 0.763 |
| vWAg | 245.6 ± 107.8% | 240.5 ± 109.0% | 0.498 |
| vWFAct/vWFAg | 0.82 ± 0.15 | 0.84 ± 0,16 | 0.300 |
| Factor VIII | 214.6 ± 73.4% | 204.5 ± 77.7% | 0.124 |

Table S3. Values of vWFAct/vWFAg, vWFAg, vWFAct and Factor VIII at baseline and four weeks follow-up in respect of MV mean gradient

|  | **MV mean gradient <4 mmHg (n=43)** | | | **MV mean gradient ≥ 4 mmHg**  **(n=33)** | | |
| --- | --- | --- | --- | --- | --- | --- |
|  | Baseline | 4 weeks follow-up | p-value | baseline | 4 weeks follow-up | p-value |
| vWFAct/vWFAg±SD | 0.87±0.13 | 0.90±0.13 | 0.182 | 0.78±0.17 | 0.78±0.18 | 0.875 |
| vWFAg±SD, (%) | 224.35±96.9 | 213.7±73.0 | 0.395 | 267.9±120.1 | 267.6±141.2 | 0.977 |
| vWFAct±SD, (%) | 194.3±83.6 | 193.5±73.5 | 0.944 | 200.0±79.1 | 195.3±87.1 | 0.516 |
| Factor VIII±SD, (%) | 209.84±70.8 | 200.3±80.3 | 0.267 | 216.2±80.5 | 204.2±81.8 | 0.346 |

MV mitral valve, SD standard deviation

Table S4. Values of vWFAct/vWFAg, vWFAg, vWFAct and Factor VIII at baseline and four weeks follow-up with mild residual MR and low post MV gradient

|  | **MV mean gradient <4 mmHg & residual MR=1**  **(n=28)** | | |
| --- | --- | --- | --- |
|  | Baseline | 4 weeks follow-up | p-value |
| vWFAct/vWFAg±SD | 0.88±0.14 | 0.90±0.14 | 0.444 |
| vWFAg±SD, (%) | 226.1±108.1 | 219.3±77.9 | 0.713 |
| vWFAct±SD, (%) | 198.8±96.4 | 200.1±81.7 | 0.938 |
| Factor VIII±SD, (%) | 216.6±68.6 | 200.8±84.2 | 0.176 |

Table S5. Values of vWFAct/vWFAg, vWFAg, vWFAct and Factor VIII at baseline and four weeks follow-up in respect of MR etiology

|  | Primary MR (n=24) | | | Secondary MR (n=61) | | |
| --- | --- | --- | --- | --- | --- | --- |
|  | Baseline | 4 weeks follow-up | p-value | baseline | 4 weeks follow-up | p-value |
| vWFAct/vWFAg±SD | 0.76±0.16 | 0.76±0.14 | 0.798 | 0.85±0.14 | 0.87±0.16 | 0.149 |
| vWFAg±SD, (%) | 250.8±128.1 | 245.6±128.8 | 0.509 | 243.6±99.8 | 238.5±101.2 | 0.616 |
| vWFAct±SD, (%) | 182.2±71.1 | 175.6±66.5 | 0.302 | 202.3±82.2 | 202.1±81.5 | 0.985 |
| Factor VIII±SD, (%) | 210.6±74.8 | 191.7±63.2 | 0.095 | 216.2±73.4 | 209.6±82.6 | 0.410 |

Table S6. Values of vWFAct/vWFAg, vWFAg, vWFAct and Factor VIII at baseline and four weeks follow-up

|  | baseline (n=85) | 4 weeks follow-up (n=85) | p-value |
| --- | --- | --- | --- |
| vWFAct/vWFAg±SD | 0.82±0.15 | 0.84±0.16 | 0.300 |
| vWFAg±SD, (%) | 245.6±107.8 | 240.5±109.0 | 0.498 |
| vWFAct±SD, (%) | 196.6±79.3 | 194.6±78.1 | 0.763 |
| Factor VIII±SD, (%) | 214.6±73.4 | 204.5±77.7 | 0.124 |

Table S7. Values of vWFAct/vWFAg, vWFAg, vWFAct and Factor VIII in respect of residual mitral regurgitation after TMVR

|  | Residual MR I  (n=47) | Residual MR II  (n=28) | Residual MR ≥ III  (n=6) | p-value |
| --- | --- | --- | --- | --- |
| vWFAct/vWFAg±SD | 0.87±0.15 | 0.82±0.18 | 0.78±0.13 | 0.280 |
| vWFAg±SD, (%) | 240.3±113.8 | 238.0±108.3 | 225.5±91.4 | 0.953 |
| vWFAct±SD, (%) | 205.5±92.0 | 181.3±53.6 | 172.4±60.3 | 0.342 |
| Factor VIII±SD, (%) | 204.8±89.3 | 203.0±60.8 | 195.0±77.1 | 0.960 |

Table S8. Values of vWFAct/vWFAg, vWFAg, vWFAct and Factor VIII in respect of LV function

|  | baseline | | | 4 weeks follow-up | | |
| --- | --- | --- | --- | --- | --- | --- |
|  | EF ≤50% (n=38) | EF >50%  (n=47) | p-value | EF ≤50% (n=38) | EF >50%  (n=47) | p-value |
| vWFAct/vWFAg±SD | 0.85±0.15 | 0.81±0.15 | 0.223 | 0.85±0.17 | 0.83±0.15 | 0.564 |
| vWFAg±SD, (%) | 232.4±69.4 | 256.3±130.7 | 0.311 | 229.6±89.8 | 249.3±122.5 | 0.411 |
| vWFAct±SD, (%) | 192.5±54.7 | 199.9±95.1 | 0.668 | 186.5±58.1 | 201.2±91.2 | 0.390 |
| Factor VIII±SD, (%) | 215.464.3 | 214.0±80.7 | 0.930 | 207.6±72.0 | 202.1±82.6 | 0.747 |

Table S9. Values of vWFAct/vWFAG, vWFAg, vWFAct and Factor VIII in respect of the implanted devices

|  | NT/NTR  (n=69) | XTR/XTR&NTR/PASCAL  (n=16) | p-value |
| --- | --- | --- | --- |
| vWFAct/vWFAg±SD | 0.84±0.17 | 0.81±0.13 | 0.462 |
| vWFAg±SD, (%) | 242.9±116.0 | 230.3±73.3 | 0.681 |
| vWFAct±SD, (%) | 198.1±84.7 | 179.5±36.5 | 0.392 |
| Factor VIII±SD, (%) | 203.1±82.8 | 210.6±51.6 | 0.732 |

Table S10. Values of vWFAct/vWFAg, vWFAg, vWFAct and Factor VIII in respect of the number of implanted devices

|  | Implantation of 1 clip (n=45) | Implantation of 2 clips (n=38) | Implantation of 3 clips (n=2) | p-value |
| --- | --- | --- | --- | --- |
| vWFAct/vWFAg±SD | 0.86±0.13 | 0.81±0.18 | 0.75±0.23 | 0.254 |
| vWFAg±SD, (%) | 221.4±94.8 | 258.3±122.3 | 331.0±60.8 | 0.152 |
| vWFAct±SD, (%) | 190.5±86.5 | 197.1±69.5 | 241.4±30.6 | 0.648 |
| Factor VIII±SD, (%) | 198.8±85.8 | 209.9±69.0 | 230.0±58.0 | 0.731 |
